# Combining corticosteroid therapies during preterm ventilation: balancing pulmonary benefits and neuroinflammatory risks

**DOI:** 10.1038/s41390-025-04239-y

**Published:** 2025-06-16

**Authors:** Abdul Razak, Atul Malhotra

**Affiliations:** 1 Department of Paediatrics, Monash University, Melbourne, VIC Australia; 2 Monash Newborn, Monash Children’s Hospital, Melbourne, VIC Australia; 3 The Ritchie Centre, Hudson Institute of Medical Research, Melbourne, VIC Australia

Bronchopulmonary dysplasia (BPD) remains a major complication of extreme prematurity, contributing significantly to long-term pulmonary morbidity and adverse neurodevelopmental outcomes. Despite advances in antenatal corticosteroids and postnatal surfactant therapy, BPD rates persist to be high in the most preterm infants, necessitating innovative postnatal interventions. Early clinical trials of intratracheal budesonide combined with surfactant suggested a potential reduction in BPD incidence, sparking enthusiasm.^1^ However, this enthusiasm has been tempered by recent evidence from a large international trial (PLUSS), which found no reduction in BPD or death among infants born <28 weeks’ gestation treated with budesonide mixed with surfactant.^2^ These neutral results have led to critical questions regarding the utility of such adjunctive therapeutic approaches, particularly in extremely preterm populations. In this context, the study by Grzych and colleagues in this issue of *Paediatric Research* provides a timely investigation into whether combining budesonide with another corticosteroid, hydrocortisone, could enhance pulmonary protection and mitigate the neuroinflammatory consequences of mechanical ventilation.^3^

Using a preterm lamb model at 125 days gestation (equivalent to ~28 weeks in humans), the investigators studied the effects of intratracheal budesonide and systemic hydrocortisone—alone and combined—in animals pretreated with antenatal betamethasone. Newborn lambs (*n* = 8 per group) received surfactant and were randomized to four ventilated groups: no additional treatment, endotracheal budesonide, intravenous hydrocortisone, or both drugs; a fifth group (*n* = 7) served as unventilated controls. All were subjected to an injurious ventilation strategy for 15 minutes using high pressures, zero PEEP, and 100% oxygen. While this model reliably induces lung injury for comparison, it diverges from modern neonatal practices that use minimally invasive and lung-protective ventilation, limiting its direct clinical relevance. Budesonide (0.25 mg/kg) was delivered intratracheally to target the lungs directly, whereas hydrocortisone (1 mg/kg) was given intravenously. Given budesonide’s 20–25-fold greater potency and the differing administration routes, these nominal doses are also not directly comparable, complicating interpretation of their relative pulmonary effects.^4^

The findings of the study were both informative and unexpected. Budesonide delivered with surfactant resulted in improved lung compliance, enhanced ventilation efficiency, and lower oxygenation indices compared with controls. It also attenuated pro-inflammatory cytokine expression in the lung and liver and was associated with reduced histological lung injury. In contrast, systemic hydrocortisone alone offered minimal benefit to pulmonary physiology, despite its favourable effect on blood pressure. Notably, when hydrocortisone was combined with budesonide, the pulmonary improvements conferred by budesonide were diminished, suggesting that the two agents may interact antagonistically in this context. The brain-specific findings were of particular concern: hydrocortisone, whether administered alone or in combination with budesonide, markedly increased astrocyte activation in both the cortex and periventricular white matter. Additionally, hydrocortisone uniquely induced microglial activation, an effect not observed with budesonide alone. These findings indicate that, in this study, even a low systemic dose of hydrocortisone triggered brain inflammation—a response not seen with budesonide—raising concern about hydrocortisone’s potential impact on early brain development.

Two key messages emerge from this study. Firstly, the combination of budesonide and hydrocortisone did not demonstrate synergism—in fact, the pulmonary benefits seen with budesonide alone appeared diminished when hydrocortisone was added. This suggests a possible antagonistic interaction between the two agents, likely due to receptor-level competition. Budesonide is a potent glucocorticoid and has minimal mineralocorticoid activity.^4^ In contrast, hydrocortisone is a much weaker glucocorticoid that also activates mineralocorticoid receptors. It is possible that hydrocortisone competes with budesonide at the glucocorticoid receptor, thereby reducing budesonide’s pulmonary effects without providing significant anti-inflammatory benefit itself. However, this receptor-level competition seems less likely given budesonide’s approximately 195-fold greater binding affinity for the receptor compared to hydrocortisone.^4^ Other explanations, such as differences in receptor activation or downstream signalling pathways, may better explain the observed interaction.

Secondly, even a single systemic dose of hydrocortisone was sufficient to induce glial activation in the brain within four hours, raising concerns about the short-term neuroinflammatory consequences of such exposure in the immediate postnatal period. This neuroinflammatory response may be attributed to the activation of mineralocorticoid receptors by hydrocortisone, which is known to promote proinflammatory pathways in the developing brain. These observations contrast with findings from clinical trials in humans, where hydrocortisone has not been linked to adverse neurodevelopmental outcomes, even at substantially higher doses. For example, the National Institute of Child Health and Human Development Neonatal Research Network hydrocortisone trial administered a cumulative dose of 18 mg/kg over 10 days to ventilator-dependent extreme preterm infants in the second and third postnatal weeks,^5^ while the Prevention of Bronchopulmonary Dysplasia by Early Low-Dose Hydrocortisone in Extremely Preterm Infants (PREMILOC) trial used 8.5 mg/kg cumulatively over the first ten days as prophylaxis,^6^ and and the Systemic Hydrocortisone To Prevent Bronchopulmonary Dysplasia (SToP-BPD) trial administered a cumulative dose of 72.5 mg/kg over a 22-day tapering schedule to very low birth weight infants who were ventilator-dependent at 7–14 days postnatal age.^7^ None of the studies reported neurodevelopmental harm, despite employing doses significantly greater than those used in the present lamb study.^5–7^ This discrepancy may reflect species-specific responses to corticosteroids and differences in timing and duration of exposure, particularly the acute administration in the animal model compared to prolonged exposure in clinical trials.

These findings also underscore a broader principle in translational therapeutics: agents that share mechanistic pathways do not necessarily act additively. Indeed, in some instances, they may counteract one another’s effects. A parallel example is found in the High-Dose Erythropoietin for Asphyxia and Encephalopathy (HEAL) trial,^8^ in which the combination of erythropoietin and therapeutic hypothermia failed to show benefit in neonatal encephalopathy, possibly due to converging mechanisms leading to diminished efficacy. The current study’s implications extend beyond corticosteroids, serving as a reminder that combination therapies require careful mechanistic evaluation, especially when both agents target overlapping molecular pathways.

Finally, it is crucial to acknowledge the inherent complexity of BPD pathogenesis. The condition results from a convergence of antenatal inflammation, ventilator-induced lung injury, oxidative stress, infection, and disrupted alveolar and vascular development. Consequently, a single postnatal intervention—whether budesonide, hydrocortisone, or both—is unlikely to fully address the multifactorial nature of the disease. For instance, the Azithromycin Therapy for Prevention of Chronic Lung Disease of Prematurity (AZTEC) trial,^9^ which evaluated azithromycin for BPD prevention, also failed to demonstrate a definitive benefit, underscoring the challenges of targeting such a complex condition with a single agent. Furthermore, BPD evolves over time, often involving multiple inflammatory and mechanical insults extending well beyond the immediate postnatal period. Therefore, brief or isolated anti-inflammatory therapies may be inadequate to significantly alter its progression.

In conclusion, this well-executed preclinical study raises important questions about the interplay between different corticosteroids used in the neonatal period. While budesonide alone demonstrated short-term pulmonary and systemic anti-inflammatory effects, the addition of hydrocortisone did not enhance and may have diminished these benefits. Furthermore, hydrocortisone exposure, even at a low dose and brief duration, was associated with early markers of neuroinflammation. Though the translational relevance of these findings requires cautious interpretation, particularly given the contrasting outcomes of clinical trials, the study highlights the need for rigorous evaluation of combination therapies. As neonatal research continues to explore adjunctive strategies to prevent BPD, this work serves as a crucial reminder that more is not always better—and that therapeutic synergy must be demonstrated, not assumed.
